# The impact of gut microbiota changes on the intestinal mucus barrier in burned mice: a study using 16S rRNA and metagenomic sequencing

**DOI:** 10.1093/burnst/tkad056

**Published:** 2023-12-19

**Authors:** Xule Zha, Sen Su, Dan Wu, Panyang Zhang, Yan Wei, Shijun Fan, Qianying Huang, Xi Peng

**Affiliations:** Clinical Medical Research Center, Southwest Hospital, Third Military Medical University (Army Medical University), Gaotanyan Street, Shapingba District, Chongqing, 400038, China; Clinical Medical Research Center, Southwest Hospital, Third Military Medical University (Army Medical University), Gaotanyan Street, Shapingba District, Chongqing, 400038, China; Clinical Medical Research Center, Southwest Hospital, Third Military Medical University (Army Medical University), Gaotanyan Street, Shapingba District, Chongqing, 400038, China; Clinical Medical Research Center, Southwest Hospital, Third Military Medical University (Army Medical University), Gaotanyan Street, Shapingba District, Chongqing, 400038, China; Clinical Medical Research Center, Southwest Hospital, Third Military Medical University (Army Medical University), Gaotanyan Street, Shapingba District, Chongqing, 400038, China; Clinical Medical Research Center, Southwest Hospital, Third Military Medical University (Army Medical University), Gaotanyan Street, Shapingba District, Chongqing, 400038, China; Clinical Medical Research Center, Southwest Hospital, Third Military Medical University (Army Medical University), Gaotanyan Street, Shapingba District, Chongqing, 400038, China; Clinical Medical Research Center, Southwest Hospital, Third Military Medical University (Army Medical University), Gaotanyan Street, Shapingba District, Chongqing, 400038, China; State Key Laboratory of Trauma, Burns and Combined Injury, Southwest Hospital, Third Military Medical University (Army Medical University), Chongqing, 400038, China

**Keywords:** Burn injury, Gut microbiota, Intestinal mucus, 16S rRNA, Metagenomic sequencing

## Abstract

**Background:**

The gut microbiota is a complex ecosystem that plays a critical role in human health and disease. However, the relationship between gut microbiota and intestinal damage caused by burns is not well understood. The intestinal mucus layer is crucial for maintaining intestinal homeostasis and providing a physiological barrier against bacterial invasion. This study aims to investigate the impact of gut microbiota on the synthesis and degradation of intestinal mucus after burns and explore potential therapeutic targets for burn injury.

**Methods:**

A modified histopathological grading system was employed to investigate the effects of burn injury on colon tissue and the intestinal mucus barrier in mice. Subsequently, 16S ribosomal RNA sequencing was used to analyze alterations in the gut microbiota at days 1–10 post-burn. Based on this, metagenomic sequencing was conducted on samples collected at days 1, 5 and 10 to investigate changes in mucus-related microbiota and explore potential underlying mechanisms.

**Results:**

Our findings showed that the mucus barrier was disrupted and that bacterial translocation occurred on day 3 following burn injury in mice. Moreover, the gut microbiota in mice was significantly disrupted from days 1 to 3 following burn injury, but gradually recovered to normal as the disease progressed. Specifically, there was a marked increase in the abundance of symbiotic and pathogenic bacteria associated with mucin degradation on day 1 after burns, but the abundance returned to normal on day 5. Conversely, the abundance of probiotic bacteria associated with mucin synthesis changed in the opposite direction. Further analysis revealed that after a burn injury, bacteria capable of degrading mucus may utilize glycoside hydrolases, flagella and internalins to break down the mucus layer, while bacteria that synthesize mucus may help restore the mucus layer by promoting the production of short-chain fatty acids.

**Conclusions:**

Burn injury leads to disruption of colonic mucus barrier and dysbiosis of gut microbiota. Some commensal and pathogenic bacteria may participate in mucin degradation via glycoside hydrolases, flagella, internalins, etc. Probiotics may provide short-chain fatty acids (particularly butyrate) as an energy source for stressed intestinal epithelial cells, promote mucin synthesis and accelerate repair of mucus layer.

Highlights

- The intestinal mucus barrier is significantly disrupted in the early stages after burn injury and there are alterations in the composition of gut microbiota. Specifically, there is a notable decrease in the population of bacteria that promote mucus production, coupled with a significant increase in the population of bacteria that break down mucus.
- Following burn injury, mucus-degrading bacteria may degrade the mucus layer through pathways such as glycoside hydrolases, flagella, internalins and other means, while mucus-synthesizing bacteria may facilitate mucus restoration by promoting the production of short-chain fatty acids.

## Background

The gut microbiota refers to the microbial community in the human intestinal tract, which includes bacteria, fungi, viruses and other microorganisms, with bacteria being the main component. There are an estimated 10–100 trillion bacteria in the human gastrointestinal tract, containing >1,500 species of bacteria [1,2]. The gut microbiota co-evolves and co-exists within the human body, forming a relatively stable ecosystem that has important impacts on the immune system, metabolism, nutrition and other aspects of the host. A large amount of basic and clinical research has confirmed that the gut microbiota is closely related to the occurrence of tumors [3], metabolic diseases [4], neurological diseases [5] and inflammatory bowel disease [6]. In recent years, the relationship between gut microbiota and organ damage caused by burns, especially intestinal damage, has received increasing attention. Damage to the intestinal mucosa caused by burn stress, ischemia and hypoxia can lead to changes in the intestinal microenvironment, resulting in disorders of the diversity and abundance of gut microbiota. In turn, changes in bacteria and their metabolites can affect the intestinal function of the host, which has significant impacts on the pathophysiology and prognosis after burn injury [7,8]. There have been studies on changes in gut microbiota caused by burn injury; however, research on the interaction between gut microbiota and the intestinal mucus barrier after burn injury has not been reported.

Intestinal mucus is the first line of defense in the intestine, playing an important physiological barrier function in maintaining the stability of the gut environment and preventing the invasion of bacteria and toxins. At the same time, the mucus is also a place for bacterial colonization, and the interaction between mucus and gut microbiota is crucial for maintaining intestinal homeostasis [9,10]. Under normal physiological conditions, the outer layer of mucus in the colon not only provides a colonization site for symbiotic bacteria, but also serves as a source of nutrition for certain bacteria. In contrast, the inner layer of mucus adheres closely to intestinal epithelial cells and has a more compact structure to prevent harmful microorganisms from invading [11]. Conversely, the gut microbiota can also affect the development and outcome of diseases by regulating the synthesis and degradation of mucus. Studies have found that mucin-degrading bacteria such as Ruminococcus and Bacteroides are significantly increased in the intestine of patients with inflammatory bowel disease [12,13]. In addition, pathogenic bacteria such as *Vibrio cholerae* and *Escherichia coli* can also degrade mucin to destroy the mucus layer, eventually leading to inflammation [14,15]. In contrast, *Lactobacillus reuteri* can increase the thickness of mucus in mice, thereby alleviating colitis induced by dextran sulfate sodium [16,17]. In recent years, there has been growing interest in a bacterium known as *Akkermansia muciniphila*, which has shown a remarkable ability to both break down mucus and stimulate mucus production. This bacterium has the unique capability to utilize mucin as an energy source when the body is experiencing malnutrition; therefore, it possesses the ability to degrade mucus [18]. Additionally, studies have revealed that when the colonic mucus layer is compromised due to a high-fat diet, *A. muciniphila* can also promote mucin synthesis and aid in mucus repair [19]. Therefore, the interplay between gut microbiota and intestinal mucus has gradually become a research hotspot in the regulation of intestinal homeostasis in disease states.

Although several studies have confirmed disruption of the gut microbiota composition in severe burns using 16S ribosomal RNA (rRNA) gene sequencing [8,20–22], it is still unclear whether microbiota can affect the synthesis and degradation of intestinal mucus, due to the limitations of research methods. Metagenomic sequencing is a technology for the deep analysis of microbial communities based on 16S rRNA. Unlike 16S rRNA sequencing, which mainly focuses on the composition and diversity of microbial communities, metagenomic sequencing can provide comprehensive information on bacterial genes, metabolism and function. Therefore, in this study, we use both methods to analyze changes in the gut microbiota post-burn and explore the mechanism by which the gut microbiota affects the synthesis and degradation of intestinal mucus. The results showed that mucin-degrading bacteria (including symbiotic and pathogenic bacteria) may degrade mucin through glycoside hydrolases, internalins and flagella, thereby exacerbating the destruction of mucus after burn injury. In contrast, short-chain fatty acids (SCFAs), derived from probiotics such as Lactobacillus and Bifidobacterium, may not only provide energy for the host under stress conditions but also promote mucus repair after burn injury. Our study provides a theoretical basis for finding new therapeutic targets for burn injury and provides ideas for exploring new burn treatment drugs and methods.

## Methods

### Animals

Healthy adult male BALB/c mice (6–8 weeks) were purchased from HFK Bioscience Co., Ltd (Beijing, China). The animals were kept in a specific pathogen-free environment at 25 ± 2°C under a photoperiod of 12 h light/12 h dark. Notably, the animals were acclimated to the environment for 1 week before the experiment.

### Experimental models

In this study, we used a burn injury model as previously described [8]. Briefly, male BALB/c mice were randomized into the following two groups: the burn group and the control group. The dorsal surface of anesthetized mice was shaved. Mice in the burn group were immersed in 90.0°C water for 10 s to cause 30% total body surface area (TBSA) burn. TBSA was calculated by the Meeh formula [23]. Lactated Ringer’s solution (1.5 ml kg^−1^%TBSA^−1^) and buprenorphine (1 mg/kg body weight) were used for fluid resuscitation and analgesia after burn injury, respectively. The mice in the burn group were housed individually in sterile cages. The same experimental procedure was performed on control mice, except that the control mice were exposed to 37°C water.

### Histopathology

The mice were euthanized by cervical dislocation. Then, the abdomen of each mouse was aseptically dissected with scissors and forceps, and the distal colon was obtained. The tissue samples were fixed in 5 ml of methanol-Carnoy’s fixative for a minimum of 3 h at room temperature and embedded in paraffin. The tissue was cut into 5 μm sections by a paraffin slicer (Leica, Wetzlar, Germany), and then stained with hematoxylin and eosin following the manufacturer’s instructions. The slices were examined by light microscopy (Olympus, Tokyo, Japan). The histological examination was performed by a scoring system as previously validated and described, as follows: tissue damage, 0 (none) to 3 (extensive mucosal damage); crypt architecture, 0 (normal) to 5 (crypt abscesses); inflammatory cell infiltration, 0 (occasional infiltration) to 3 (transmural infiltration); goblet cell depletion, 0 (normal) to 3 (>50% depletion). Crypt lengths (0–4) were measured using CellSens software (Olympus, Tokyo, Japan).

The mucus and goblet cells in paraffin-embedded colon sections were analyzed by Alcian blue/periodic acid Schiff (AB/PAS) staining. The samples were stained with Alcian blue staining solution for 15 min and rinsed with water until colorless, and then stained with 0.5%–1% periodic acid solution for 15 min and rinsed with water for 5 min. Thirdly, the tissues were stained in Schiff reagent at room temperature for 30 min in the dark and rinsed for 5 min. Finally, the tissues were dehydrated with gradient ethanol (75, 95 and 100% ethanol, respectively) for 5 min and xylene transparent for 5 min. The slices were examined by light microscopy (Olympus, Tokyo, Japan).

### Fluorescence *in situ* hybridization

Fluorescence *in situ* hybridization was performed as previously described in detail [24]. Briefly, the tissue sections were directly labeled with the Cyanine 3-conjugated universal bacterial probe EUB338 (5′-GCTGCCTCCCGTAGGAGT-3′, Invitrogen). After probing and hybridization, the slices were restained with 4′,6-diamidino-2-phenylindole. Coimmunostaining with anti-mucin 2 (MUC2) (ab272692, Abcam) was performed.

### 16S rRNA sequencing and bioinformatics analysis

Fresh fecal pellets were respectively collected from 10 burn mice and 10 control mice over a period of 10 days. The samples were immediately frozen in liquid nitrogen and then transferred into a −80°C cryogenic freezer for cryopreservation until DNA extraction. Microbial DNA was extracted using a QIAamp Fast DNA Stool Mini Kit (Qiagen, Valencia, CA, USA) according to the manufacturer’s instructions. The qualified DNA samples were stored at −80°C. The V3-V4 region of the bacterial 16S rRNA gene was amplified using primer pairs 338F (5′-ACTCCTACGGGAGGCAGCAG-3′) and 806R (5′-GGACTACHVGGGTWTCTAAT-3′) by a Bio-Rad T100 PCR thermocycler (Bio-Rad, Hercules, USA). The PCR product was extracted from a 2% agarose gel and purified using a DNA gel extraction kit (Omega, Atlanta, USA). Purified amplicons were pooled in equimolar amounts and paired-end sequenced on an Illumina MiSeq PE300 platform/NovaSeq PE250 platform (Illumina, San Diego, USA) according to the standard protocols by Majorbio Bio-Pharm Technology Co. Ltd (Shanghai, China).

A DADA2 plug-in (or Deblur plug-in) in the QIIME2 process was used to reduce the noise of the optimized sequence after quality control concatenation to obtain the amplicon sequence variants (ASVs). Based on the ASV information, alpha diversity indices including Chao richness and Shannon index were calculated with Mothur v1.30.1. The similarity among the microbial communities in different samples was determined by principal component analysis (PCA) based on Bray–Curtis dissimilarity using the Vegan v2.5–3 package. Python-2.7 software was used to analyze the community composition of different groups at phylum and genus levels. The significance level of species abundance differences was evaluated based on the Wilcoxon rank-sum test using R-3.3.1 (stat) software.

Linear discriminant analysis (LDA) effect size (LefSe) analysis was conducted using the relative abundance of indicator microorganisms in burned and control mice at phylum to genus level (http://huttenhower.sph.harvard.edu/galaxy/root?tool_id=lefse_upload). Taxonomy levels were incorporated into all groupings within the text files, which were formatted according to the required structure. An ‘all-against-all’ strategy was adopted for multiclass analysis, with the factorial Kruskal–Wallis test and pairwise Wilcoxon signed-rank test for all groupings set at a Monte-Carlo (alpha = 0.05). The results are presented as cladograms and included taxa with an LDA threshold of >3.5 for the analyses.

### Metagenomic sequencing and bioinformatics analysis

Total genomic DNA was fragmented to an average size of ~400 bp using Covaris M220 (Gene Company Limited, China) for paired-end library construction. A paired-end library was constructed using NEXTFLEX Rapid DNA-Seq (Bioo Scientific, Austin, TX, USA). Adapters containing the full complement of sequencing primer hybridization sites were ligated to the blunt-end of fragments. Then, metagenomic sequencing was performed on an Illumina NovaSeq 6000 platform (Illumina Inc, San Diego, CA, USA) at Majorbio Bio-Pharm Technology Co., Ltd (Shanghai, China) according to the manufacturer’s protocols. The low-quality reads (length < 50 bp or with a quality value < 20 or having *N* bases) were removed from the raw data by fastp, and high-quality paired-end reads and single-end reads were retained. Metagenomics data were assembled using MEGAHIT v1.1.2. Contigs with a length ≥ 300 bp were selected as the final assembly result, and the contigs were used for further gene prediction and annotation. Open reading frames from each assembled contig were predicted using Prodigal v2.6.3. The predicted open reading frames with a length ≥ 100 bp were retrieved and translated into amino acid sequences. A non-redundant gene catalog was constructed using CD-HIT v4.7 with 90% sequence identity and 90% coverage. High-quality reads were aligned to the non-redundant gene catalogs to calculate gene abundance with 95% identity using SOAPaligner software.

Representative sequences of non-redundant gene catalog were aligned to the NR database with an e-value cutoff of 1e^−5^ using Diamond for taxonomic annotations. Kyoto Encyclopedia of Genes and Genomes (KEGG) annotation was conducted using Diamond against the KEGG database (http://www.genome.jp/keeg/) with an e-value cutoff of 1e^−5^.

A circos map was generated at pathway level 1 from the KEGG database using Circos-0.67-7 (http://circos.ca/).

### Statistical analysis

GraphPad Prism v.9.5.1 and SPSS 18.0 software were used for other statistical analyses and graphing. The quantitative data were subjected to normality test by the Shapiro–Wilk test, and Levene’s test was used to determine the homogeneity of variance. Normally distributed data are presented as the mean ± standard deviation (SD) and non-normal distributed data are presented as median (P25 to P75) (lower quartiles to upper quartiles). In order to exclude the time factor, the method of matching the burn group and the control group at the same time point was adopted to study the changes of gut microbiota after burn in mice. Comparisons between the two groups were performed using independent samples t-test or Wilcoxon rank sum test. In all analyses, a two-tailed *p* < 0.05 was considered to indicate a statistically significant difference; ^*^p < 0.05, ^*^^*^p < 0.01 and ^*^^*^^*^p < 0.001.

## Results

### Burn injury results in colonic damage and disruption of the mucus barrier in mice

**Figure 1:**
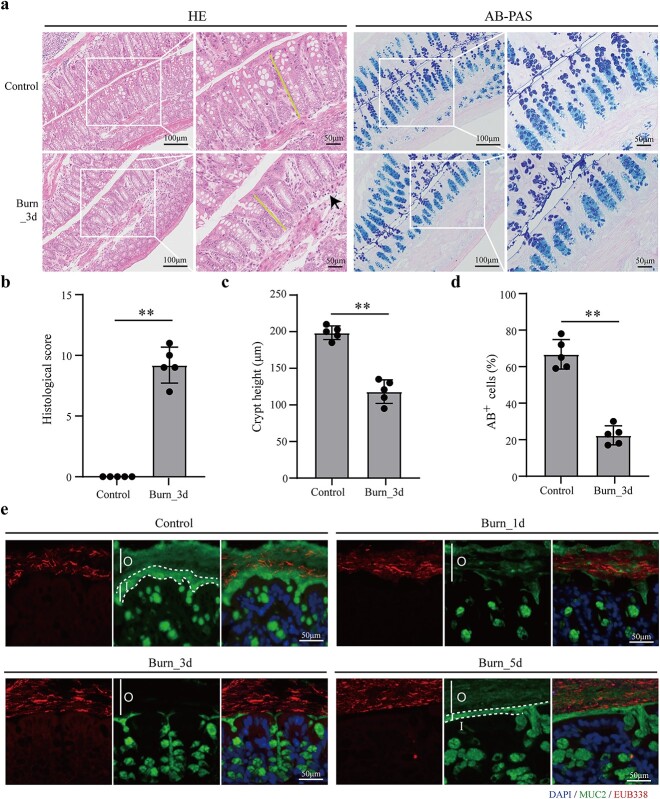
The effect of burn injury on colonic tissue and intestinal mucus barrier. (**a**) hematoxylin and eosin (HE) and Alcian blue/periodic acid Schiff (AB/PAS) staining of the distal colon in mice on day 3 after burn injury. The yellow line indicates the height of the crypt. Aggregation of basal lymphocytes is indicated by a black arrow. Scale bars: 50 or 100 μm. (**b**) The histological score of the colonic tissue was calculated from colon sections (n = 5 per group); ^*^^*^p < 0.01. (**c**) The height of colonic crypts was measured in mice from the control and burn groups (n = 5 per group); ^*^^*^p < 0.01. (**d**) The number of goblet cells was counted based on AB/PAS-stained colon sections (n = 5 per group); ^*^^*^p < 0.01. (**e**) Immunostaining of colon sections was performed using antibodies against mucin 2 (MUC2) (green) and fluorescence *in situ* hybridization with a bacterial 16S ribosomal RNA (rRNA) gene probe (EUB338, red) at days 1, 3 and 5 post-burn. The white dashed line delineates the inner mucus layer (I) and the outer mucus layer (O). Nuclei were stained with 4′,6-diamidino-2-phenylindole (blue). Scale bar: 50 μm

In this study, we utilized an enhanced histopathological grading system to assess the impact of burn injury on the colons of mice. The histological examination of hematoxylin and eosin staining revealed that the colonic crypts were shortened, collapsed and infiltrated by inflammatory cells on day 3 post-burn in mice (Figure 1a–c), indicating colonic tissue damage induced by burn injury. The AB/PAS staining results demonstrated a significant decrease in colonic mucin content on day 3 following burn injury in mice, which suggests that burn injury disrupts the colonic mucus barrier, resulting in subsequent intestinal damage (Figure 1a, d). To further evaluate the effects of burns on the mucus layer of the colon, immunostaining with MUC2 antibody (green) was conducted on the distal colon of mice at days 1, 3 and 5 post-burn. Additionally, universal 16S rRNA probes (red) were employed for *in situ* hybridization analysis to examine the spatial distribution of bacteria within the tissue. The results showed that the colon mucus layer of normal mice was divided into outer and inner layers, with bacteria existing only in the outer layer while the inner layer was bacteria-free (Figure 1e). The inner mucus layer of the colon was damaged, and the structure of the outer mucus layer became looser, allowing bacteria to penetrate the inner layer on day 1 post-burn (Figure 1e). The mucus layer of the colon was destroyed completely, and bacteria could adhere directly to the colonic epithelium on day 3 post-burn (Figure 1e). However, the mucus layer of the colon was regenerated both internally and externally, allowing for effective isolation of bacteria in the outer mucus layer on day 5 post-burn (Figure 1e). These findings suggest that the integrity of the mucous barrier in mice was compromised during the initial 3 days post-burn and began to gradually recover from the fifth day postburn.

### Diversity analysis of gut microbiota in mice after burn injury

**Figure 2:**
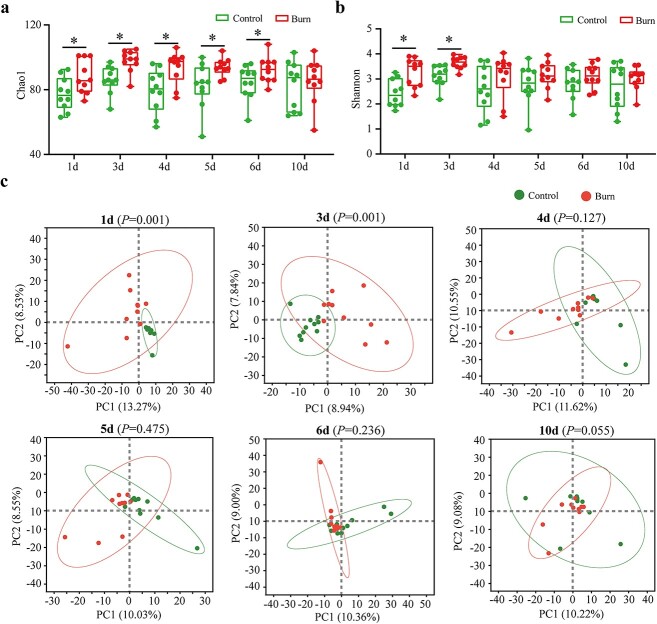
Diversity of gut microbiota at days 1, 3, 4, 5, 6 and 10 post-burn. (**a**, **b**) Chao1 and Shannon index were used to measure alpha diversity post-burn; ^*^p < 0.05. (**c**) Principal component analysis (PCA) analysis of gut microbiota post-burn. PC1 and PC2 represent the two selected principal coordinate components, and the percentage represents the contribution value of the principal coordinate components to the difference in sample composition. Each circle represents a sample. 1d, 3d, 4d, 5d, 6d and 10d represent days 1, 3, 4, 5, 6 and 10, respectively

Intestinal mucus serves as the niche for colonization of gut microbiota, and mutual modulation between the mucus and microbiota is essential to maintain intestinal homeostasis. Our findings demonstrated that the integrity of the mucus barrier may be compromised by burn injury, while the barrier gradually recovers over time. To assess the changes of gut microbiota during this period, we collected fecal samples from mice at days 1–10 after burn injury for 16S rRNA sequencing. Alpha diversity analysis showed a significant increase in Chao index from day 1 to day 6 post-burn (Figure 2a), as well as a significant elevation in Shannon index from day 1 to day 3 post-burn (Figure 2b), indicating a marked increase in both community abundance and diversity following burn injury. PCA revealed good overall sample repeatability between the burn and control groups. Significant differences were observed between the burn and control groups during the first 3 days following burn injury (*p* = 0.001). However, no significant inter-group difference was observed 5 days after burn injury (*p* > 0.05), suggesting a gradual normalization of the gut microbiota as burn injury progressed (Figure 2c).

### Taxonomic composition analysis of the gut microbiota in mice after burn injury

**Figure 3:**
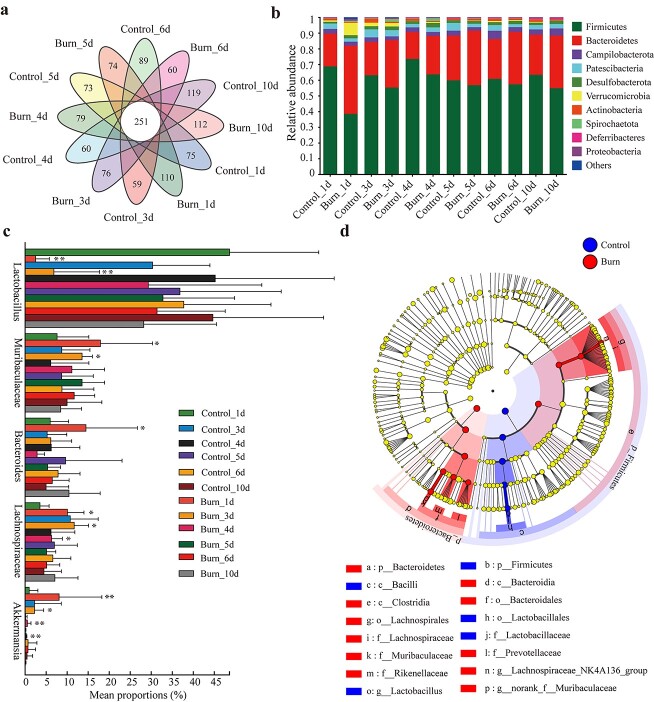
Severe burn altered the composition of gut microbiota. (**a**) Venn diagram of the composition of amplicon sequence variants (ASVs) in gut microbiota. (**b**) Bar-plot analysis shows relative abundance of the top ten microbial communities at the phylum level. The *x-*axis represents the group information and the *y-*axis represents relative abundance of specific bacterial phyla. (**c**) Relative abundance of microbial community at the genus level; ^*^p < 0.05, ^*^^*^p < 0.01. (**d**) Linear discriminant analysis (LDA) effect size (LefSe) analysis of gut microbiota composition differences comparing burn and control mice. Different colored nodes represent the significantly enriched microbial taxa in the corresponding groups that have a significant impact on inter-group differences. LDA score > 3.5

The Venn diagram in Figure 3a shows the shared and unique ASVs in the control and burn groups. A significant increase in the number of ASVs was observed from the first to fourth day after burn injury, followed by a gradual decrease in ASVs (Figure 3a). The results indicated that burn injury led to changes in the quantity of gut microbiota. Additionally, the abundance of gut microbiota in mice after burn injury was analyzed at the phylum level. The community composition analysis revealed that Firmicutes and Bacteroidetes in the control group accounted for ~68.8 and 20.85% of the total bacteria, respectively (Figure 3b, Figure S1, see online supplementary material). On the first day after burn injury, there was a significant decrease in the abundance of Firmicutes to 38.48% and a significant increase in the Bacteroidetes to 43.39%. These changes gradually returned to normal levels compared to the control group over the following 3 days after the injury (Figure S1). Verrucomicrobia is present at a very low abundance, accounting for only ~0.9% of the total microbiota in normal mice. Compared to the control group, the abundance of Verrucomicrobia rapidly increased to 7.88% 1 day after burn injury, followed by a trend of decreasing and then increasing over the next 3–5 days (Figure 3a, Figure S1). Further observation of burned mice at the genus level revealed that Lactobacillus, Muribaculaceae, Bacteroides and Lachnospiraceae were the top four species in terms of relative abundance (Figure S2, see online supplementary material). Lactobacillus significantly decreased from day 1 to day 3 in burned mice, while Muribaculaceae significantly increased during the same period. Lachnospiraceae showed a significant increase in abundance from day 1 to day 5 after burning, while Bacteroides showed a significant increase in abundance on the first day after burning (Figure 3c). Our findings indicated that the composition of gut microbiota in burned mice was disrupted from day 1 to day 3 after burn injury, followed by a gradual restoration toward normalcy 3 days after burn injury. LEfSe analysis was performed to identify biomarkers within the gut microbiota of the control and burn groups. Lactobacillus was higher in the control group, while Muribaculaceae, Bacteroides and Lachnospiraceae were enriched significantly in the burn group (Figure 3d). Additionally, the abundance of Akkermansia in burned mice changed in accordance with that of the Verrucomicrobia, to which it belongs (Figure 3c, Figure S3, see online supplementary material). As is commonly recognized, Akkermansia has been closely linked to the formation and degradation of mucus, while Lactobacillus, as one of the most commonly used probiotics, plays a crucial role in promoting mucus production. Thus, we speculate that gut microbiota including Akkermansia and Lactobacillus may play an important role in the disruption and restoration of gut mucus after burn injury.

### The gut microbiota is implicated in the degradation and restoration of intestinal mucus following burn injury

**Figure 4:**
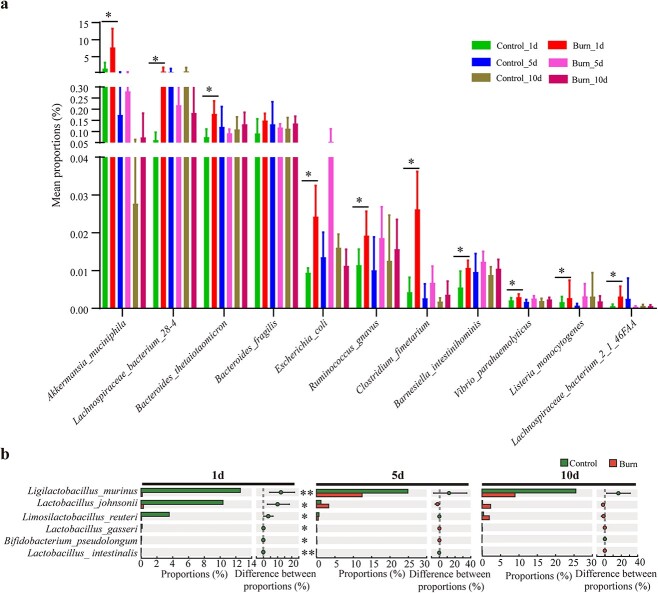
The relative abundance of mucus-associated bacteria was significantly altered post-burn. (**a**) Changes in the relative abundance of mucus-degrading bacteria (commensal and pathogenic bacteria) in mice 1, 5 and 10 days after burn injury. (**b**) Changes in the relative abundance of mucus-synthesis bacteria (probiotics) in mice 1, 5 and 10 days after burn injury. 1d, 5d and 10d represent days 1, 5 and 10, respectively. ^*^ *p*< 0.05, ^*^^*^p < 0.01

Samples from the first, fifth and tenth day after burn injury were subsequently selected for metagenomic sequencing to explore the impact of gut microbiota on the intestinal mucus barrier at the gene and functional levels. The compositional changes in the gut microbiota in burned mice at the phylum and genus levels, as determined by metagenomic sequencing data (Figure S4, see online supplementary material), were similar to the results obtained from 16S rRNA sequencing. Next, we analyzed the abundance changes in bacteria associated with mucin synthesis and degradation at the species level based on metagenomic data. Our findings revealed a noteworthy increase in the abundance of *A. muciniphila* on post-burn day 1 compared to that of the control group, and the increased level returned to normal on days 5 and 10 (Figure 4a). We propose that this elevation in abundance is primarily attributable to the initial stress response triggered on the first day after burn injury. This response prompts the significant secretion of a large amount of stored mucus from goblet cells, which serves to resist unfavorable factors. Further analysis indicates that the abundance of Akkermansia exhibited a dynamic trend within 3–5 days after burn injury (Figure 3c, Figure S3). We postulate that the reduction in *A. muciniphila* abundance on day 3 after burn injury may be attributed to the disruption of the intestinal mucus layer. As the mucus content continues to decrease, it may stimulate the proliferation of *A. muciniphila* to promote mucin synthesis and maintain the stability of the intestinal mucus.

In addition, a range of symbiotic and pathogenic bacteria also contribute to mucus degradation. Our results indicate that pathogenic bacteria, such as *E. coli*, *Clostridium fimetarium*, *Vibrio parahaemolyticus* and *Listeria monocytogenes*, as well as symbiotic bacteria including *Barnesiella intestinihominis*, *Ruminococcus gnavus*, *Bacteroides thetaiotaomicron*, *Bacteroides fragilis*, *Lachnospiraceae bacterium 28–4* and *Lachnospiraceae bacterium 2_1_46FAA*, all significantly increased in abundance on the first day after burn injury when participating in mucus degradation (Figure 4a). Further research showed a significant reduction in the abundance of *Lactobacillus intestinalis*, *Lactobacillus johnsonii*, *Lactobacillus gasseri*, *Ligilactobacillus murinus*, *Limosilactobacillus reuteri* of the Lactobacillus and *Bifidobacterium pseudolongum* of the Bifidobacterium on day 1 after burn injury, compared to that of the control group. However, the abundance of these bacteria subsequently recovered to normal levels by days 5 and 10 after burn injury (Figure 4b). Our findings indicate that the abundance of bacteria involved in promoting mucin synthesis may be regulated by the mucus content in the intestinal lumen. We speculate that on the first day after burn, a significant influx of mucus occurs in the intestine, which inhibits the growth of these bacteria. However, as the disease progresses and the mucus content gradually decreases, there is an increase in the abundance of these bacteria, which stimulates mucin synthesis.

To further verify that the abundance changes in bacteria associated with mucin synthesis and degradation may be related to the integrity of the mucus layer, based on the changes in the mucus layer of burned mice (Figure 1e), the control 1 day, control 5 day and burn 5 day groups, with an intact inner mucus layer, were classified as the inner mucus group (IMG), while the burn 1 day group, with the absence of the inner mucus layer, was classified as the inner mucus absent group (IMAG). Six bacterial species from the mucus-associated bacteria described above were randomly selected to compare their relative abundance within the IMG and the IMAG. Statistical analysis showed a significant increase in the relative abundance of *A. muciniphila*, *C. fimetarium*, *B. thetaiotaomicron* and *L. bacterium 2_1_46FAA* in the IMAG, while *B. pseudolongum* and *L. johnsonii* were significantly decreased (Figure S5, see online supplementary material), further suggesting the possible correlation between their abundance changes and the mucus layer.

### Bacteria may degrade intestinal mucus through various mechanisms after burn injury

**Figure 5:**
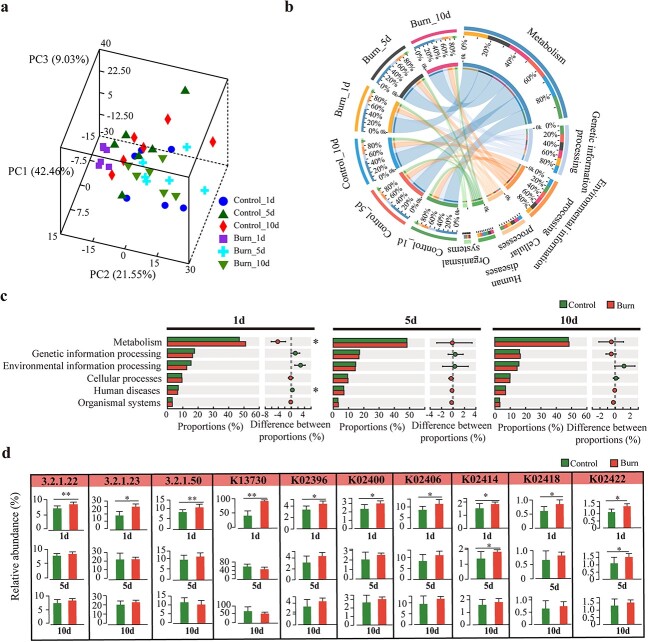
The impact of burn injury on gut microbiota function was revealed by metagenomic sequencing. (**a**) Principal component analysis (PCA) analysis of control mice and burn mice based on Kyoto Encyclopedia of Genes and Genomes (KEGG) pathway level 1. PC1, PC2 and PC3 represent the top three principal component factors that affect the functional composition of the sample, and the percentage represents the contribution value of the principal coordinate components to the difference in sample functional composition. (**b**) Circos plot illustrating the correlation between different samples and their corresponding functions. The left half circle represents the sample group, and the right half circle represents the distribution of functions in different samples at the clustering level. (**c**) Alterations in biological metabolic pathways at the first level of the KEGG database after burn injury in mice. (**d**) Changes in the abundance of glycoside hydrolases, internalins and flagella-related enzymes after burn injury. 1d, 5d and 10d represent days 1, 5 and 10, respectively; ^*^p < 0.05, ^*^^*^p < 0.01

Next, we performed KEGG enrichment analysis based on the results of metagenomic sequencing to investigate the underlying mechanisms by which gut microbiota affects mucus layer. The functions in the KEGG database are divided into the following three levels: pathway level 1, pathway level 2 and pathway level 3. The PCA analysis based on KEGG pathway level 1 showed that the samples on the first day after burn injury were noticeably separated from the other samples, indicating that the function of gut microbiota was significantly affected the first day after burn injury (Figure 5a). Biological metabolic pathways are classified into six categories at the first level of the KEGG database. Among them, metabolism and human diseases were significantly impacted 1 day after burn injury in mice, whereas they recovered to normal at days 5 and 10 (Figure 5b, c). To explore the mechanism of bacterial mucus degradation in mice after burn injury, we focused on these two metabolic pathways. One of the important ways in which bacteria degrade mucus, as reported by previous studies, is through glycoside hydrolases, including sialidases, *N*-acetylglucosaminidases and galactosidases. Further analysis revealed that the galactose metabolism (ko00052) and glycosaminoglycan degradation (ko00531) signaling pathways in the metabolism pathway were significantly affected after burn injury. Specifically, the abundance of alpha-galactosidase (3.2.1.22: K07406, K07407), beta-galactosidase (3.2.1.23: K01190, K12308) and alpha-*N*-acetylglucosaminidase (3.2.1.50: K01205) was significantly elevated in mice at day 1 after burn injury, while there was no significant difference compared to that in normal mice at days 5 and 10 post-burn (Figure 5d, Table S1, see online supplementary material). In addition, the abundance of internalin A (K13730), which in involved in mucus degradation, was significantly elevated in the bacterial invasion of epithelial cells (ko05100) signaling pathway of the human diseases metabolic pathway (Figure 5d, Table S1). The adhesion and degradation of mucins by the flagella of certain symbiotic and pathogenic microbiota in the gut have been a subject of special interest [25]. Therefore, we focused on investigating the alterations in the signaling pathways related to flagella formation after burn injury. Our data demonstrated a significant impact on flagellar assembly (ko02040) at day 1 after burn injury, with an increased abundance of flagellar biosynthesis protein FlhA (K02400), flagellin (K02406), flagellar protein FliO/FliZ (K02418), flagellar hook-length control protein FliK (K02414), hook-associated protein 1 flgK (K02396) and flagellar protein FliS (K02422) (Figure 5d, Table S1). Based on our observations, we speculate that mucin-degrading bacteria may exacerbate mucin degradation after burn injury through mechanisms involving glycoside hydrolases, internalins and flagella.

### The gut microbiota may provide energy to the host and facilitate post-burn mucus repair through SCFAs

**Figure 6:**
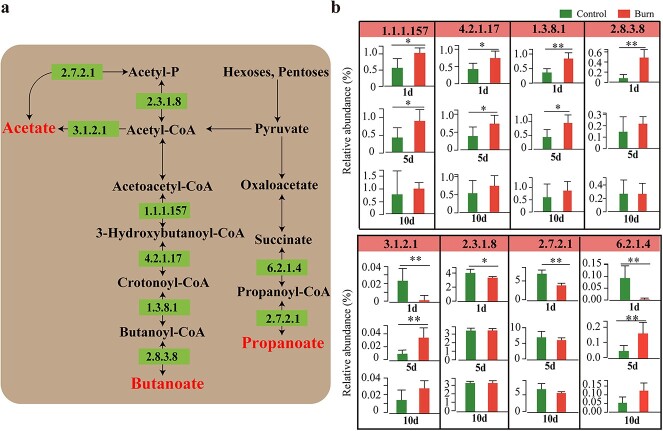
Effects on microbiota-derived short-chain fatty acids (SCFAs) synthesis after burn injury. (**a**) The synthesis pathway of acetate, propionate and butyrate in the gut microbiota. (**b**) Changes in the abundance of enzymes responsible for acetate, propionate and butyrate synthesis after burn injury. 1d, 5d and 10d represent days 1, 5 and 10, respectively; ^*^p < 0.05, ^*^^*^p < 0.01

Currently, there is a limited understanding of the mechanisms by which probiotics promote mucus repair. One of the key mechanisms by which probiotics promote mucus secretion and increase mucin thickness is through SCFAs (acetate, butyrate, propanoate etc.), the important metabolic products of the gut microbiota [26]. Butyrate serves as the primary energy source for intestinal epithelial cells [27]. Inter-group differential analysis revealed that the abundance of key enzymes involved in the butyrate synthesis pathway, including 3-hydroxybutyryl-CoA dehydrogenase (1.1.1.157: K00074), enoyl-CoA hydratase (4.2.1.17: K01692, K01715), butyryl-CoA dehydrogenase (1.3.8.1: K00248) and acetate CoA-transferase (2.8.3.8: K01034, K01035, K19709), significantly increased on the first and fifth days after burn injury. (Figure 6a and b, Table S2, see online supplementary material). This finding was consistent with changes in the abundance of butyrate-producing bacteria. Our results suggest that the host may need to synthesize butyrate to provide a large amount of energy at days 1 and 5 post-burn, and butyrate synthesis returns to normal on day 10 postburn. Under anaerobic conditions, pyruvate is converted into acetyl coenzyme A (acetyl-CoA) by pyruvate formate lyase, which is then metabolized to acetate through the phosphotransacetylase (Pta)-acetate kinase (AckA) pathway. Additionally, acetyl-CoA hydrolase catalyzes the production of acetate from acetyl-CoA. We found that the abundance of key enzymes involved in the Pta-AckA pathway, including phosphate acetyltransferase (2.3.1.8: K15024, K13788) and acetate kinase (2.7.2.1: K00925), as well as acetyl-CoA hydrolase (3.1.2.1: K01067), was significantly reduced on day 1 post-burn (Figure 6a and b, Table S2). Meanwhile, succinyl-CoA synthetase (6.2.1.4: K01899, K01900) and acetate kinase, which are involved in the synthesis of propionate, showed similar trends (Figure 6a and b, Table S2). This indicates that the synthesis of acetate and propionate was significantly reduced on day 1 post-burn but returned to normal levels at days 5 and 10 postburn. Overall, we speculate that the host is in a stressed state on day 1 postburn and requires a significant amount of butyrate to maintain normal intestinal homeostasis. During the recovery phase (days 5 and 10 postburn), acetate, propanoate and butyrate may promote mucus repair.

## Discussion

Intestinal mucus is the first barrier of the intestinal mucosa and damage to the mucus barrier after burns is an important factor in aggravating intestinal injury. It is currently believed that the bidirectional regulatory effect of mucus and gut microbiota is crucial to maintaining intestinal homeostasis [28]. In this study, the changes in gut microbiota, especially mucus-related microbiota, at different stages of burns were comprehensively analyzed using 16S rRNA gene sequencing and metagenomic sequencing, and the mechanisms that affect intestinal mucus were explored. Our findings demonstrated that the composition of gut microbiota is altered significantly following burn injury, with changes in the relative abundance of Firmicutes, Bacteroidetes, Verrucomicrobia, Proteobacteria and Actinobacteria, and most notably Firmicutes and Bacteroidetes. Furthermore, some symbiotic and pathogenic bacteria may degrade mucin through glycoside hydrolase, internalins and flagella, while probiotics such as Lactobacillus and Bifidobacterium may promote mucus synthesis through SCFAs.

Firmicutes and Bacteroidetes are the most predominant classes in the gut microbiota [29,30]. Our results indicated a significant decrease in the abundance of Firmicutes and a significant increase in Bacteroidetes in mice on day 1 following burn injury, while the abundance of both phyla had largely returned to normal levels on day 3 after burn injury, suggesting that severe burn injury can disrupt the gut microbiota, and that the gut microbiota gradually recovers to normal levels as burn injury progresses. The studies by Feng *et al*. and Beckmann *et al*. are consistent with our findings, however, the former found that dysbiosis of the microbiota can last for up to 3–5 days after burn injury [8,20]. Furthermore, we found that the abundance of Verrucomicrobia was significantly increased by nearly 9-fold the first day after burn injury, despite its low abundance under normal physiological conditions. Akkermansia, a representative genus of Verrucomicrobia, is a unique probiotic. Studies have demonstrated its ability to utilize mucin from host mucus to provide energy under conditions of malnutrition, showcasing its characteristic of mucin degradation [31]. Additionally, Akkermansia can promote mucin synthesis to accelerate the repair of mucosal damage caused by a high-fat diet-induced disruption of the colonic mucus layer [19,32]. In our findings, Akkermansia initially increased, followed by a decrease, and then an increase again from day 1 to day 5 after burn injury in mice. We hypothesized that goblet cells are under stress on the first day following burn injury in mice, leading to the excessive secretion of mucin stored in cells and the consequent accumulation of mucin in the intestinal lumen. At that time, the abundance of Akkermansia greatly increases to degrade the excessive mucin and to maintain the stability of the intestinal mucus layer. Three days post-burn, there was a notable decrease in mucins in the intestinal lumen, which subsequently caused a reduction in Akkermansia abundance. The continuous decrease in mucus content may stimulate the gradual proliferation of Akkermansia to promote mucin synthesis. The experimental results confirmed that Akkermansia may play a dual role in degrading mucin and promoting mucin synthesis according to changes in intestinal mucin levels, which is crucial for maintaining the stability of the intestinal mucus barrier after burn injury.

Further studies found that the abundance of symbiotic bacteria and pathogenic bacteria was significantly increased in the early stage after burn injury and participated in the degradation of intestinal mucus. Png *et al*. validated that Ruminococcus can utilize MUC2 as the sole carbon source by degrading it [33]. In addition, the colonization of *B. thetaiotaomicron* in mice is dependent on mucin [34]. Early studies proved that *B. fragilis* can bind intestinal mucin [35]. In addition to commensal bacteria, pathogenic bacteria mentioned in this study can directly or indirectly regulate goblet cell function and mucin expression, thereby inhibiting mucin synthesis or promoting mucin degradation [12,36,37]. The mechanisms underlying the degradation of mucins by commensal and pathogenic bacteria may include various factors such as glycoside hydrolases, flagella, internalins and others. Published data have shown that *A. muciniphila, B. thetaiotaomicron, Bifidobacterium bifidum,* and others can degrade mucins through glycoside hydrolases [38]. Furthermore, some pathogenic bacteria, such as *L. monocytogenes*, can synthesize a toxin protein called internalins, which interacts with MUC2 to disrupt mucin structure [39]. In addition, several microbes have evolved to adhere to and penetrate the mucin layer using extracellular appendages such as flagella [25]. Studies have found that flagella and flagellins of enteropathogenic *E. coli* and enterohemorrhagic *E. coli* can bind to intestinal mucin and penetrate the mucin layer to infect epithelial cells [40]. Our results showed a significant increase in the abundance of flagellins and internalins after burn injury, indicating that some microbes may disrupt intestinal mucus through these factors. Thus, we propose that disrupted gut microbiome after burn injury may degrade mucin and disrupt the intestinal mucus barrier through various factors such as glycoside hydrolases, flagella and internalins.

The decrease in probiotic abundance after burns is another important factor leading to damage of the intestinal mucus barrier. In addition to *A. muciniphila*, the abundance of Lactobacillus and Bifidobacterium also significantly decreased. Studies have found that these two probiotics can inhibit the excessive proliferation and adhesion of pathogenic bacteria to intestinal epithelial cells through competitive inhibition, thereby suppressing gut derived infections [41]. Additionally, administering these two probiotics separately or in combination can alleviate the intestinal inflammatory response and promote mucin synthesis [42]. They are currently the most widely used bacterial strains in clinical treatment. Studies have revealed that facilitating the synthesis of SCFAs is one of the pivotal mechanisms by which probiotics maintain the intestinal mucus barrier [43–45]. SCFAs, also known as volatile fatty acids, are fatty acids that generally have six carbon atoms or fewer and include acetate, propionate and butyrate [46]. Acetate is considered the most abundant SCFAs in animal bodies, while butyrate is the principal source of energy for intestinal epithelial cells [47]. Our findings indicated that the alterations in these three SCFAs exhibited distinct patterns. Specifically, on the first day after burns, the abundance of butyrate-producing bacteria was significantly augmented, and the abundance of enzymes implicated in butyrate biosynthesis, including butyryl-CoA dehydrogenase, 3-hydroxybutyryl-CoA dehydrogenase and enoyl-CoA hydratase, was markedly elevated. This tendency persisted until the fifth day after burn injury and the abundances subsequently returned to baseline levels on day 10. In contrast, the activities of enzymes responsible for acetate synthesis, namely, acetyl-CoA hydrolase, phosphate acetyltransferase and acetate kinase, were conspicuously diminished on day 1 post-burn, which persisted until day 5 before returning to normal. Furthermore, the crucial enzymes involved in propionate biosynthesis exhibited a similar trend. This observation implies that the exigency of butyrate synthesis in intestinal epithelial cells outstrips that of acetate and propionate following burns. Reducing acetate and propionate synthesis to ensure butyrate synthesis is beneficial for improving intestinal epithelial cell energy metabolism. The aforementioned results indicate the existence of a tightly regulated symbiotic mechanism between microbes and the host. Gut bacteria have the ability to accurately regulate the abundance of bacterial populations and relevant metabolic enzymes in response to the host’s demands, which helps to alleviate damage to intestinal epithelial cells and maintain the integrity of the intestinal mucus barrier. In addition to providing the necessary energy to intestinal epithelial cells, butyrate can promote mucus synthesis and maintain the intestinal mucus barrier through various direct or indirect pathways. Butyrate has been reported to accelerate the transcription of MUC2 by promoting histone acetylation of the MUC2 promoter [48]. Additionally, butyrate can also accelerate the repair of the damaged intestinal mucus barriers by activating the Wingless/Integrated-Extracellular Signal Regulated Kinase signaling pathway to promote MUC2 synthesis [27]. Through this study, the changes in SCFAs after burn injury were clarified, and the differences in the synthesis of butyrate, acetate and propionate by gut microbiota and their underlying pathophysiological significance were discovered. These findings highlight the importance of focusing on the supplementation of butyrate, rather than acetate and propionate, when administering SCFA preparations to patients with burn injury.

In this study, we predicted the function and mechanism of mucin-related bacteria through 16S rRNA and metagenomic sequencing. In future studies we will isolate bacterial strains from the mouse intestine that promote mucin synthesis and explore the mechanism of these bacterial strains in regulating mucin synthesis. These studies will lay the foundation for further optimization and development of microbial preparations that can effectively promote mucin synthesis and maintain the integrity of the intestinal mucus barrier.

## Conclusions

To summarize, colonic injury and intestinal mucus barrier disruption are caused by burns in mice, accompanied by dysbiosis of gut microbiota. Various mucin-degrading bacteria, including symbiotic and pathogenic bacteria, may degrade mucins through glycoside hydrolases, flagella, internalins etc., leading to damage to the intestinal mucus barrier. However, probiotics such as Lactobacillus and Bifidobacterium may provide energy to intestinal epithelial cells under stress conditions via SCFAs (especially butyrate), which promote mucin synthesis and accelerate the repair of damaged intestinal mucus barrier.

## Supplementary Material

supplementary_tkad056

## Abbreviations

AB/PAS, Alcian blue/periodic acid Schiff; Acetyl-CoA: Acetyl coenzyme A; AckA: Acetate kinase; ASVs: Amplicon sequence variants; IMAG: Inner mucus absent group; IMG: Inner mucus group; KEGG: Kyoto Encyclopedia of Genes and Genomes; LDA: Linear discriminant analysis; LEfSe: Linear discriminant analysis effect size; MUC2: Mucin 2; PCA: Principal component analysis; Pta: Phosphotransacetylase; rRNA: Ribosomal RNA; SCFA: Short-chain fatty acids; TBSA: Total body surface area.

## References

[1] Lozupone CA, Stombaugh JI, Gordon JI, Jansson JK, Knight R. Diversity, stability and resilience of the human gut microbiota. Nature. 2012;489:220–30. 22972295 10.1038/nature11550 PMC3577372

[2] Yang XX, Guo YX, Chen C, Shao B, Zhao LY, Zhou QB, et al. Interaction between intestinal microbiota and tumour immunity in the tumour microenvironment. Immunology. 2021;164:476–93. 34322877 10.1111/imm.13397 PMC8517597

[3] Tilg H, Adolph TE, Gerner RR, Moschen AR. The intestinal microbiota in colorectal cancer. Cancer Cell. 2018;33:954–64. 29657127 10.1016/j.ccell.2018.03.004

[4] Aron-Wisnewsky J, Warmbrunn MV, Nieuwdorp M, Clément K. Metabolism and metabolic disorders and the microbiome: the intestinal microbiota associated with obesity, lipid metabolism, and metabolic health-pathophysiology and therapeutic strategies. Gastroenterology. 2021;160:573–99. 33253685 10.1053/j.gastro.2020.10.057

[5] Ma Q, Xing C, Long W, Wang HY, Liu Q, Wang RF. Impact of microbiota on central nervous system and neurological diseases: the gut-brain axis. J Neuroinflammation. 2019;16:53. 30823925 10.1186/s12974-019-1434-3 PMC6397457

[6] Weingarden AR, Vaughn BP. Intestinal microbiota, fecal microbiota transplantation, and inflammatory bowel disease. Gut Microbes. 2017;8:238–52. 28609251 10.1080/19490976.2017.1290757 PMC5479396

[7] Niu M, Chen P. Crosstalk between gut microbiota and sepsis. *Burns*. Trauma. 2021;9:tkab036. 10.1093/burnst/tkab036 PMC8547143 34712743

[8] Feng Y, Huang Y, Wang Y, Wang P, Wang F. Severe burn injury alters intestinal microbiota composition and impairs intestinal barrier in mice. Burns Trauma. 2019;7:20. 31312663 10.1186/s41038-019-0156-1 PMC6610819

[9] Paone P, Cani PD. Mucus barrier, mucins and gut microbiota: the expected slimy partners? Gut. 2020;69:2232–43. 32917747 10.1136/gutjnl-2020-322260 PMC7677487

[10] Zhao Q, Maynard CL. Mucus, commensals, and the immune system. Gut Microbes. 2022;14:2041342. 35239459 10.1080/19490976.2022.2041342 PMC8903774

[11] Hansson GC . Mucins and the microbiome. Annu Rev Biochem. 2020;89:769–93. 32243763 10.1146/annurev-biochem-011520-105053 PMC8442341

[12] Cornick S, Tawiah A, Chadee K. Roles and regulation of the mucus barrier in the gut. Tissue Barriers. 2015;3:e982426. 25838985 10.4161/21688370.2014.982426 PMC4372027

[13] Pereira FC, Berry D. Microbial nutrient niches in the gut. Environ Microbiol. 2017;19:1366–78. 28035742 10.1111/1462-2920.13659 PMC5412925

[14] Johansson ME, Sjövall H, Hansson GC. The gastrointestinal mucus system in health and disease. Nat Rev Gastroenterol Hepatol. 2013;10:352–61. 23478383 10.1038/nrgastro.2013.35 PMC3758667

[15] Martens EC, Neumann M, Desai MS. Interactions of commensal and pathogenic microorganisms with the intestinal mucosal barrier. Nat Rev Microbiol. 2018;16:457–70. 29904082 10.1038/s41579-018-0036-x

[16] Ahl D, Liu H, Schreiber O, Roos S, Phillipson M, Holm L. Lactobacillus reuteri increases mucus thickness and ameliorates dextran sulphate sodium-induced colitis in mice. Acta Physiol. 2016;217:300–10. 10.1111/apha.12695 27096537

[17] Bene KP, Kavanaugh DW, Leclaire C, Gunning AP, MacKenzie DA, Wittmann A, et al. Lactobacillus reuteri surface mucus adhesins upregulate inflammatory responses through interactions with innate C-type lectin receptors. Front Microbiol. 2017;8:321. 28326063 10.3389/fmicb.2017.00321 PMC5339304

[18] Ouwerkerk JP, de Vos WM, Belzer C. Glycobiome: bacteria and mucus at the epithelial interface. Best Pract Res Clin Gastroenterol. 2013;27:25–38. 23768550 10.1016/j.bpg.2013.03.001

[19] Everard A, Belzer C, Geurts L, Ouwerkerk JP, Druart C, Bindels LB, et al. Cross-talk between Akkermansia muciniphila and intestinal epithelium controls diet-induced obesity. Proc Natl Acad Sci U S A. 2013;110:9066–71. 23671105 10.1073/pnas.1219451110 PMC3670398

[20] Beckmann N, Pugh AM, Caldwell CC. Burn injury alters the intestinal microbiome's taxonomic composition and functional gene expression. PLoS One. 2018;13:e0205307. 30289947 10.1371/journal.pone.0205307 PMC6173435

[21] Earley ZM, Akhtar S, Green SJ, Naqib A, Khan O, Cannon AR, et al. Burn injury alters the intestinal microbiome and increases gut permeability and bacterial translocation. PLoS One. 2015;10:e0129996. 26154283 10.1371/journal.pone.0129996 PMC4496078

[22] Corcione S, Lupia T, De Rosa FG. Microbiome in the setting of burn patients: implications for infections and clinical outcomes. Burns Trauma. 2020;8:tkaa033. 32821744 10.1093/burnst/tkaa033 PMC7428410

[23] Gilpin DA . Calculation of a new Meeh constant and experimental determination of burn size. Burns. 1996;22:607–11. 8982538 10.1016/s0305-4179(96)00064-2

[24] Wu D, Su S, Zha X, Wei Y, Yang G, Huang Q, et al. Glutamine promotes O-GlcNAcylation of G6PD and inhibits AGR2 S-glutathionylation to maintain the intestinal mucus barrier in burned septic mice. Redox Biol. 2023;59:102581. 36565645 10.1016/j.redox.2022.102581 PMC9800542

[25] Juge N . Microbial adhesins to gastrointestinal mucus. Trends Microbiol. 2012;20:30–9. 22088901 10.1016/j.tim.2011.10.001

[26] Deleu S, Machiels K, Raes J, Verbeke K, Vermeire S. Short chain fatty acids and its producing organisms: an overlooked therapy for IBD? EBioMedicine. 2021;66:103293. 33813134 10.1016/j.ebiom.2021.103293 PMC8047503

[27] Liang L, Liu L, Zhou W, Yang C, Mai G, Li H, et al. Gut microbiota-derived butyrate regulates gut mucus barrier repair by activating the macrophage/WNT/ERK signaling pathway. Clin Sci. 2022;136:291–307. 10.1042/CS20210778 35194640

[28] Etienne-Mesmin L, Chassaing B, Desvaux M, De Paepe K, Gresse R, Sauvaitre T, et al. Experimental models to study intestinal microbes-mucus interactions in health and disease. FEMS Microbiol Rev. 2019;43:457–89. 31162610 10.1093/femsre/fuz013

[29] Eckburg PB, Bik EM, Bernstein CN, Purdom E, Dethlefsen L, Sargent M, et al. Diversity of the human intestinal microbial flora. Science. 2005;308:1635–8. 15831718 10.1126/science.1110591 PMC1395357

[30] Qin J, Li R, Raes J, Arumugam M, Burgdorf KS, Manichanh C, et al. A human gut microbial gene catalogue established by metagenomic sequencing. Nature. 2010;464:59–65. 20203603 10.1038/nature08821 PMC3779803

[31] Belzer C, Chia LW, Aalvink S, Chamlagain B, Piironen V, Knol J, et al. Microbial metabolic networks at the mucus layer lead to diet-independent butyrate and vitamin B12 production by intestinal symbionts. MBio. 2017;8:e00770–17. 28928206 10.1128/mBio.00770-17 PMC5605934

[32] Shin NR, Lee JC, Lee HY, Kim MS, Whon TW, Lee MS, et al. An increase in the Akkermansia spp. population induced by metformin treatment improves glucose homeostasis in diet-induced obese mice. Gut. 2014;63:727–35. 23804561 10.1136/gutjnl-2012-303839

[33] Png CW, Lindén SK, Gilshenan KS, Zoetendal EG, McSweeney CS, Sly LI, et al. Mucolytic bacteria with increased prevalence in IBD mucosa augment in vitro utilization of mucin by other bacteria. Am J Gastroenterol. 2010;105:2420–8. 20648002 10.1038/ajg.2010.281

[34] Hayase E, Hayase T, Jamal MA, Miyama T, Chang CC, Ortega MR, et al. Mucus-degrading Bacteroides link carbapenems to aggravated graft-versus-host disease. Cell. 2022;185:3705–3719.e14. 36179667 10.1016/j.cell.2022.09.007 PMC9542352

[35] Huang JY, Lee SM, Mazmanian SK. The human commensal Bacteroides fragilis binds intestinal mucin. Anaerobe. 2011;17:137–41. 21664470 10.1016/j.anaerobe.2011.05.017 PMC3163789

[36] Sheikh A, Wangdi T, Vickers TJ, Aaron B, Palmer M, Miller MJ, et al. Enterotoxigenic Escherichia coli degrades the host MUC2 mucin barrier to facilitate critical pathogen-enterocyte interactions in human small intestine. Infect Immun. 2022;90:e0057221. 34807735 10.1128/iai.00572-21 PMC8853678

[37] Crowther RS, Roomi NW, Fahim RE, Forstner JF. Vibrio cholerae metalloproteinase degrades intestinal mucin and facilitates enterotoxin-induced secretion from rat intestine. Biochim Biophys Acta. 1987;924:393–402. 3297167 10.1016/0304-4165(87)90153-x

[38] Ndeh D, Gilbert HJ. Biochemistry of complex glycan depolymerisation by the human gut microbiota. FEMS Microbiol Rev. 2018;42:146–64. 29325042 10.1093/femsre/fuy002

[39] Popowska M, Krawczyk-Balska A, Ostrowski R, Desvaux M. InlL from listeria monocytogenes is involved in biofilm formation and adhesion to mucin. Front Microbiol. 2017;8:660. 28473809 10.3389/fmicb.2017.00660 PMC5397405

[40] Erdem AL, Avelino F, Xicohtencatl-Cortes J, Girón JA. Host protein binding and adhesive properties of H6 and H7 flagella of attaching and effacing Escherichia coli. J Bacteriol. 2007;189:7426–35. 17693516 10.1128/JB.00464-07 PMC2168434

[41] Li SC, Hsu WF, Chang JS, Shih CK. Combination of lactobacillus acidophilus and Bifidobacterium animalis subsp. lactis shows a stronger anti-inflammatory effect than individual strains in HT-29 cells. Nutrients. 2019;11:969. 31035617 10.3390/nu11050969 PMC6566532

[42] Kumar M, Kissoon-Singh V, Coria AL, Moreau F, Chadee K. Probiotic mixture VSL#3 reduces colonic inflammation and improves intestinal barrier function in Muc2 mucin-deficient mice. Am J Physiol Gastrointest Liver Physiol. 2017;312:G34–45. 27856417 10.1152/ajpgi.00298.2016

[43] Blaak EE, Canfora EE, Theis S, Frost G, Groen AK, Mithieux G, et al. Short chain fatty acids in human gut and metabolic health. Benef Microbes. 2020;11:411–55. 32865024 10.3920/BM2020.0057

[44] Lou X, Xue J, Shao R, Mo C, Wang F, Chen G. Postbiotics as potential new therapeutic agents for sepsis. Burns Trauma. 2023;11:tkad022. 37334140 10.1093/burnst/tkad022 PMC10271603

[45] Wang K, Zeng Q, Li X, Wang Y, Wang L, Sun W, et al. Efficacy of probiotics or synbiotics for critically ill adult patients: a systematic review and meta-analysis of randomized controlled trials. Burns Trauma. 2022;10:tkac004. 35291228 10.1093/burnst/tkac004 PMC8918756

[46] Martin-Gallausiaux C, Marinelli L, Blottière HM, Larraufie P, Lapaque N. SCFA: mechanisms and functional importance in the gut. Proc Nutr Soc. 2021;80:37–49. Koh A. 32238208 10.1017/S0029665120006916

[47] De Vadder F, Kovatcheva-Datchary P, Bäckhed F. From dietary fiber to host physiology: short-chain fatty acids as key bacterial metabolites. Cell. 2016;165:1332–45. 27259147 10.1016/j.cell.2016.05.041

[48] Hatayama H, Iwashita J, Kuwajima A, Abe T. The short chain fatty acid, butyrate, stimulates MUC2 mucin production in the human colon cancer cell line, LS174T. Biochem Biophys Res Commun. 2007;356:599–603. 17374366 10.1016/j.bbrc.2007.03.025

