# Supplementary material for: The impact of gut microbiota changes on the intestinal mucus barrier in burned mice: a study using 16S rRNA and metagenomic sequencing
Source: Burns Trauma. 2023 Dec 19;11:tkad056. doi: 10.1093/burnst/tkad056 (PMC10734567; doi:10.1093/burnst/tkad056)
Supplement: supplementary_tkad056 [file supplementary_tkad056.docx]

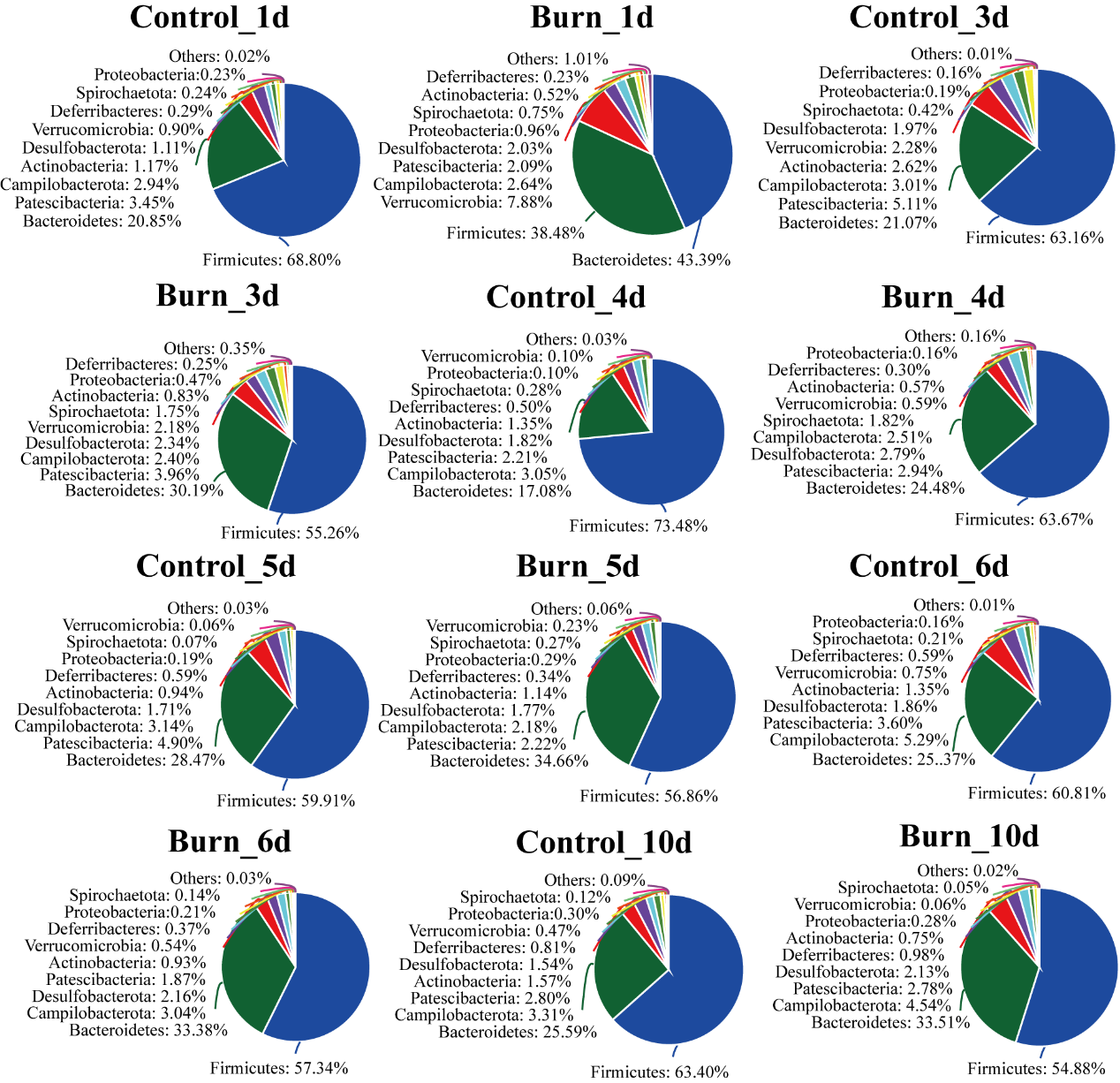


**Figure S1.** Community analysis pieplot of the top 10 microbial communities at the phylum level.


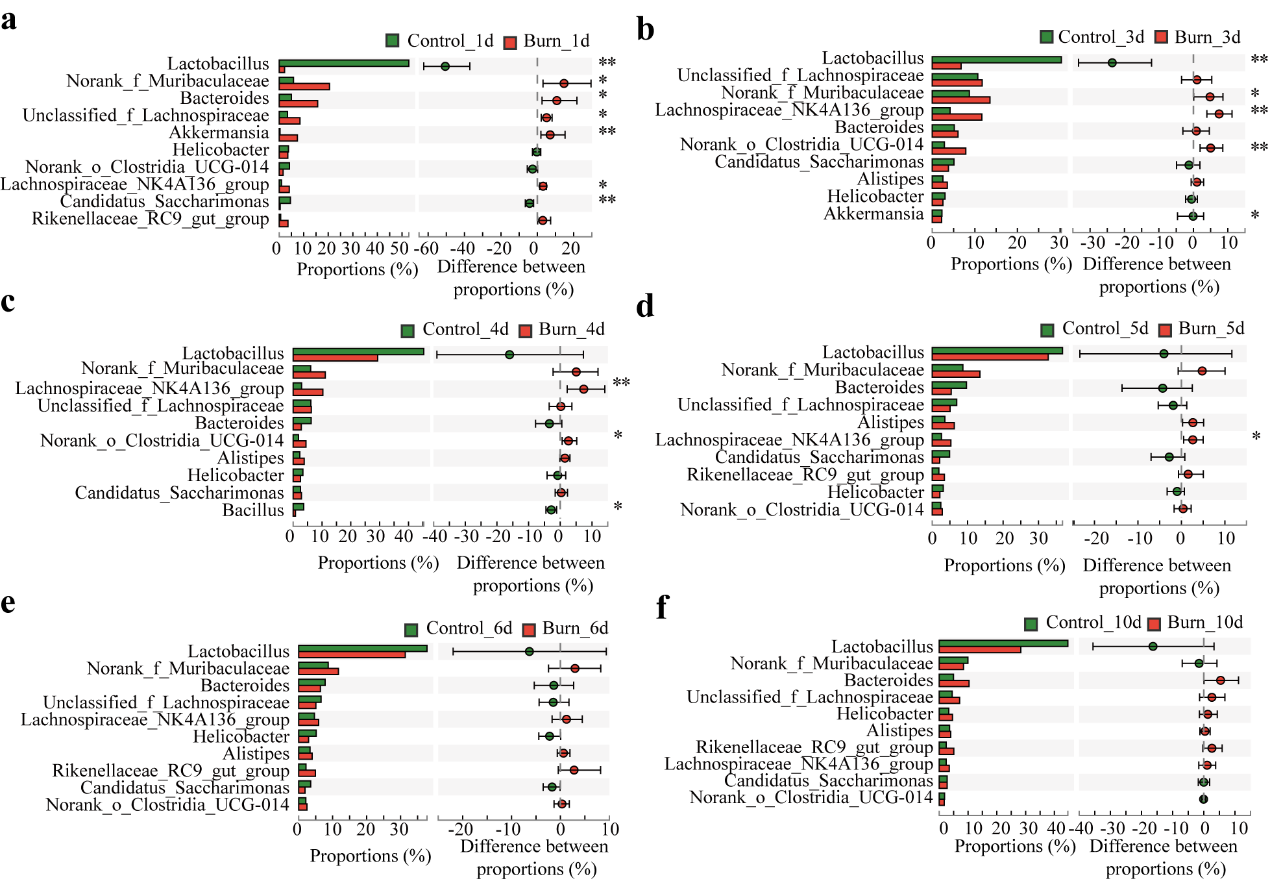


**Figure S2.** The relative abundance of the top 10 microbial communities at the genus level after burn injury in mice. (**a**) The first day following burn injury. (**b**) The third day following burn injury. (**c**) The fourth day following burn injury. (**d**) The fifth day following burn injury. (**e**) The sixth day following burn injury. (**f**) The tenth day following burn injury. *p < 0.05, **p < 0.01.


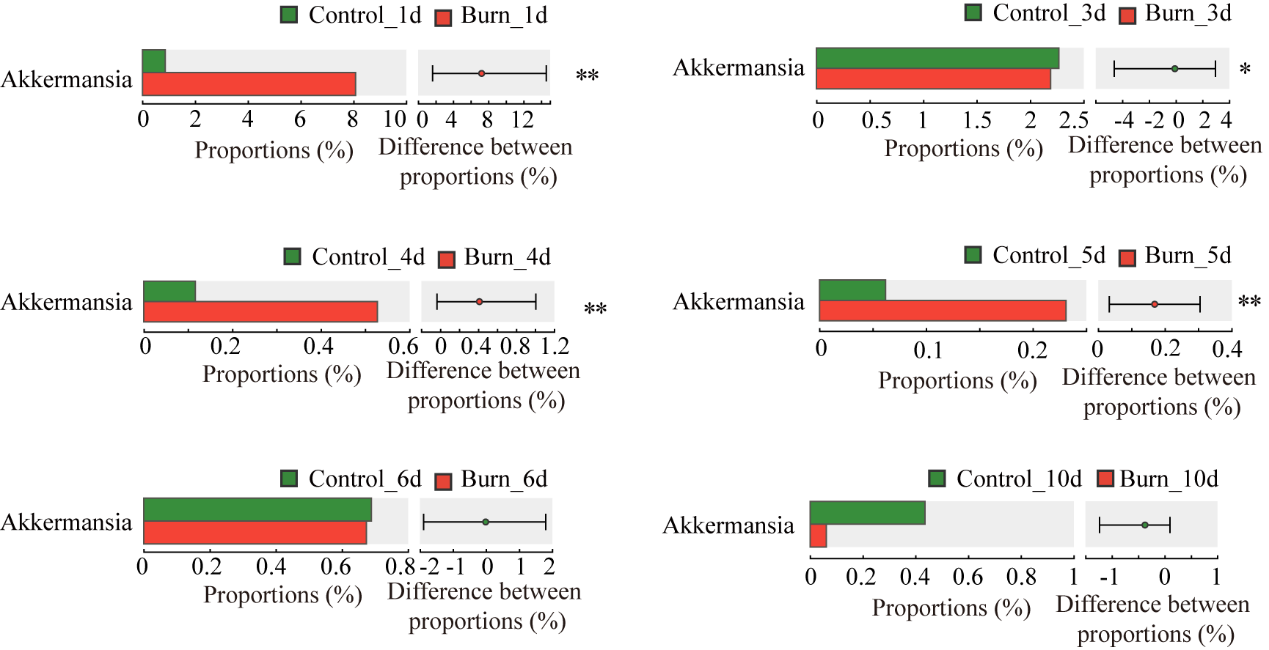


**Figure S3.** Variation in the relative abundance of Akkermansia from 1-10 days after burn injury. *p < 0.05, **p < 0.01.

**
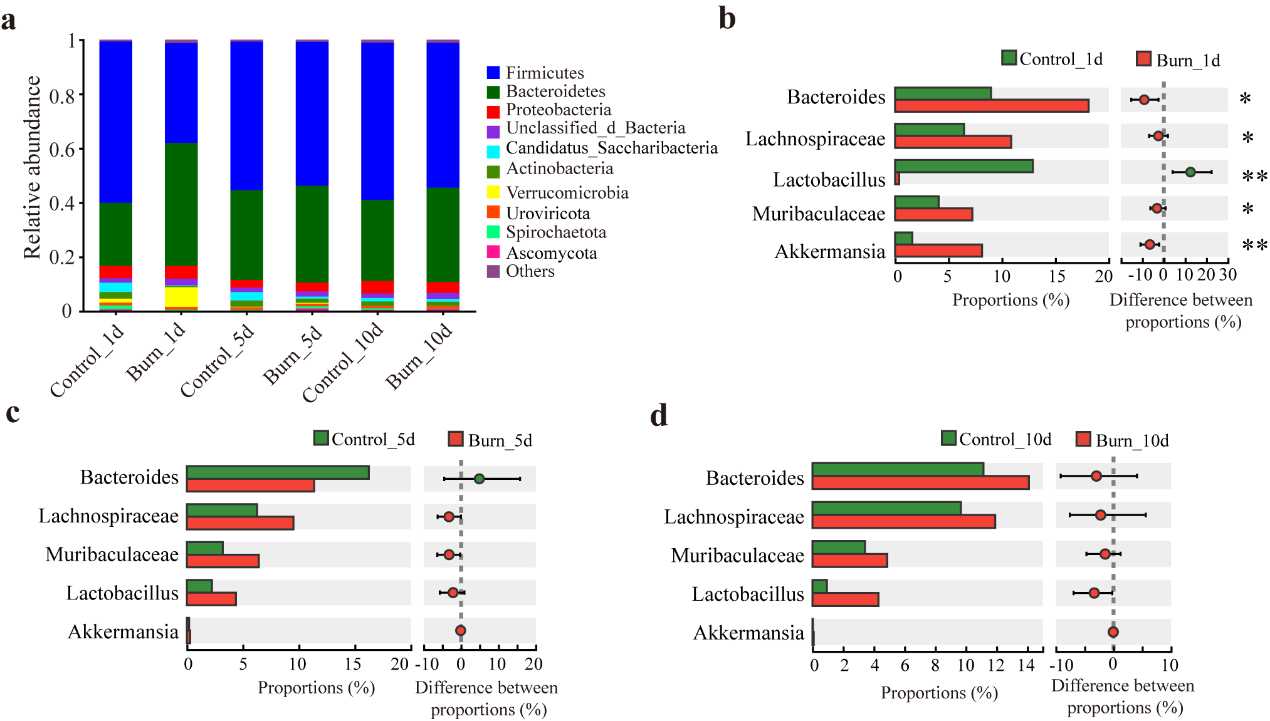
**

**Figure S4.** Compositional changes in the gut microbiota of burned mice analyzed at the phylum and genus levels using metagenomic sequencing. (**a**) The bar-plot analysis revealed the relative abundance changes of the top 10 microbial communities in burned mice at the phylum level. (**b, c, d**) The abundance of Lactobacillus, Muribaculaceae, Bacteroides, Lachnospiraceae, and Akkermansia at the genus level showed changes in mice on the first day (**b**), the fifth day (**c**), the tenth day (**d**) after burn injury. *p < 0.05, **p < 0.01.

**
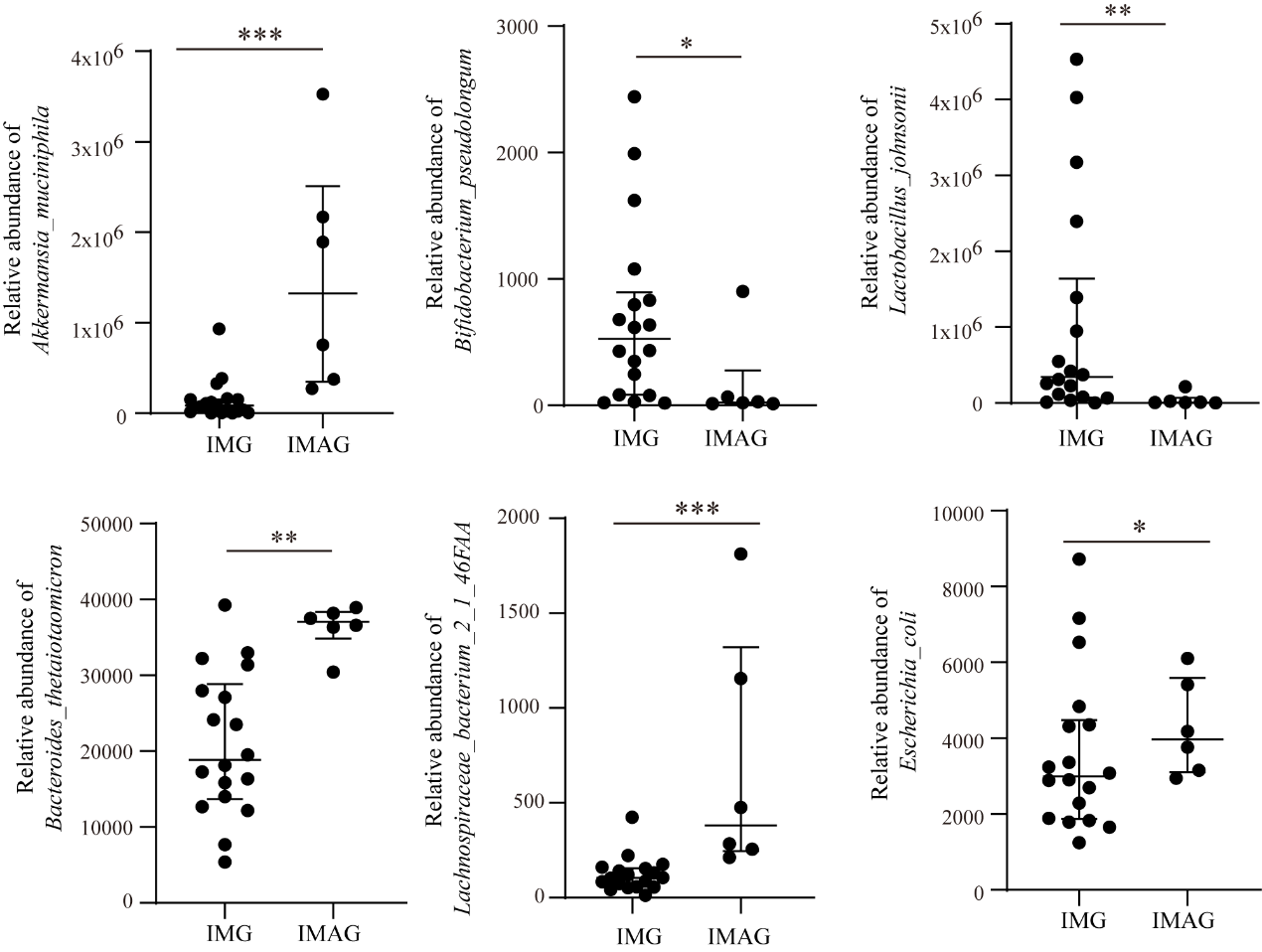
Figure S5.** The abundance changes in bacteria related to mucin synthesis and degradation in IMG and IMAG. Each black dot represents a sample, IMG contains the control_1d, control_5d, and burn_5d groups with a total of 18 samples, while IMAG contains the burn_1d group with a total of 6 samples. *p < 0.05, **p < 0.01, ***p < 0.001.

**Table S1.** The fundamental information of mucin-degrading enzymes in various KEGG pathways

| **Enzyme** | **KO** | **KEGG Name** | **KO Description** | **KEGG Pathway** |
| --- | --- | --- | --- | --- |
| EC:3.2.1.22 | K07406 | melA | alpha-galactosidase | Galactose metabolism  (ko00052) |
|  | K07407 | Gal A |  |  |
| EC:3.2.1.23 | K01190 | lacZ | beta-galactosidase |  |
|  | K12308 | lacA |  |  |
| EC:3.2.1.50 | K01205 | NAGLU | alpha-N-acetylglucosaminidase | Glycosaminoglycan degradation  (ko00531) |
| —— | K02396 | flgK | hook-associated protein 1 | Flagellar assembly (ko02040) |
| —— | K02400 | flhA | flagellar biosynthesis protein |  |
| —— | K02406 | fliC | flagellin |  |
| —— | K02418 | fliO | flagellar protein |  |
| —— | K02414 | fliK | flagellar hook-length control protein |  |
| —— | K02422 | fliS | flagellar protein |  |
| —— | K13730 | inlA | internalin A | Bacterial invasion of epithelial cells (ko05100) |

**Table S2.** The fundamental information of mucin-synthesis enzymes in various KEGG pathways

| **Enzyme** | **KO** | **KEGG Name** | **KO Description** | **KEGG Pathway** |
| --- | --- | --- | --- | --- |
| EC:1.1.1.157 | K00074 | hbd | 3-hydroxybutyryl-CoA dehydrogenase | Butanoate metabolism  （ko00650） |
| EC:4.2.1.17 | K01692 | PaaF/echA | enoyl-CoA hydratase |  |
|  | K01715 | crt | enoyl-CoA hydratase |  |
| EC:1.3.8.1 | K00248 | ACADS, bcd | butyryl-CoA dehydrogenase |  |
| EC:2.8.3.8 | K01034 | atoD | acetate CoA-transferase alpha subunit |  |
|  | K01035 | atoA | acetate CoA-transferase beta subunit |  |
|  | K19709 | ydiF | acetate CoA-transferase |  |
| EC:3.1.2.1 | K01067 | ACH1 | acetyl-CoA hydrolase | Pyruvate metabolism  （ko00620） |
| EC:2.3.1.8 | K15024 | pta | phosphate acetyltransferase |  |
|  | K13788 | pta | phosphate acetyltransferase |  |
| EC:2.7.2.1 | K00925 | ackA | acetate kinase |  |
| EC:6.2.1.4 | K01899 | LSC1 | succinyl-CoA synthetase alpha subunit | Propanoate  Metabolism  (ko00640) |
|  | K01900 | LSC2 | succinyl-CoA synthetase beta subunit |  |
